# Impacts of vaping and marijuana use on airway health as determined by exhaled breath condensate (EBC)

**DOI:** 10.1186/s12931-025-03147-3

**Published:** 2025-02-21

**Authors:** Dante E. Rojas, Mitchell M. McCartney, Eva Borras, Tristan L. Hicks, Tiffany T. Lam, Nicholas J. Kenyon, Cristina E. Davis

**Affiliations:** 1 Mechanical and Aerospace Engineering, University of California, Davis, Davis, CA USA; 2 UC Davis Lung Center, University of California, Davis, CA USA; 3 VA Northern California Health Care System, Mather, CA USA; 4 Department of Internal Medicine, University of California, Sacramento, CA USA

## Abstract

Across the United States, there is increased use of cannabis products and electronic delivery systems for cannabis products and nicotine, yet little is known about their impacts on lung health. We analyzed exhaled breath condensate of 254 participants who were non-users and users who used cannabis and tobacco products. The 132 participants reported using a product (“users”) were distributed into cohorts of tobacco products and cannabis products, with some participants following into multiple cohorts. Targeted analysis of inflammatory oxylipins found up-regulation among persons using tobacco products, while cannabis users had concentrations closer to nonusers, and often down-regulated. Untargeted screening of 403 significant metabolites found tobacco users had similar breath profiles, and that cannabis users had a similar profile that was closer to the profile of nonusers. Metabolites were significantly higher in breath of people using combustion products (tobacco and cannabis) relative to nonusers, and significantly lower in e-device users (nicotine and THC). Our work demonstrates the relative impact of e-delivery systems and cannabis products compared to traditional cigarette smoking on lung metabolic profiles.

**Supplementary Information:**

The online version contains supplementary material available at 10.1186/s12931-025-03147-3.

## Background

Tobacco and cannabis use are critical research areas for public health. While harmful effects of toxicants in conventional cigarette smoke are well documented, newer nicotine delivery devices like e-cigarettes and vapes are often marketed as safer alternatives to cigarettes, but the relatively small body of scientific literature available suggests otherwise. Meanwhile, the legalization of cannabis across the United States has spurred increased consumption, but federal prohibitions have historically prevented clinical epidemiological studies to determine the safety of marijuana use. As vaping and cannabis use continue to rise, especially among younger populations, there is an urgent need to assess the impacts of vaping and cannabis use on pulmonary health.

As cigarettes burn, combustion generates reactive oxygen species (ROS) on the order of 10^17^ molecules per puff [1] that induce peroxidation of airway cell membranes and activates epithelial signaling, producing cytokines like interleukin-8 (IL-8) that promote chronic immune cell activation and inflammation [2]. Furthermore, cigarette smoke contains 83 carcinogenic volatile organic compounds (VOCs) like benzene and acrolein [3, 4].

Evidence suggests combustion of cannabis flower may have similar effects, as it also introduces ROS to the airway, triggering inflammatory processes [5]. However, the health effects of inhaled cannabis products, especially regarding the pulmonary immune system, are still subject of investigation due to the presence of cannabinoids that modulate immune function and decrease the associated inflammation [6, 7]. Given the increasing of cannabis use, understanding its effects on airway health is critical for developing public health policies that convey risks compared to tobacco.

Marketed as safer alternatives to combustion-based cigarettes, electronic devices appeared in the early 2000s and include a variety of products such as vapes, vaporizers and electronic cigarettes. Either nicotine or tetrahydrocannabinol (THC) is dissolved in an “e-liquid” mixture of propylene glycol or vegetable glycerin, sometimes with added flavorings (5). When the user activates the device, a heater vaporizes the e-liquid to produce a plume for inhalation. Moreover, due to the variability in the design of these devices and the composition of e-liquids, questions persist about the toxicants present, the health risks to the airways, and the physiological response generated in users (7).

There is a strong body of evidence that the body response to vape exposure similar to cigarette smoke. This has been observed in cultures of human skin and lung cells, which exhibited comparable levels of cellular damage and morbidity with exposure to e-liquid relative to cigarette smoke [8]. Vape liquid stimulated production of pro-inflammatory cytokines [9] and other proteins [10, 11] in a murine model. Another murine model found that VL negatively impacted lung function to a similar degree as CS [12]. Finally, researchers have reported levels of heavy metals like lead [13] in vape liquid, in addition to toxicant VOCs like benzene, though at concentrations sometimes order of magnitude lower than cigarette smoke [14].

Thus, it is anticipated that cannabis consumption and use of nicotine- or THC-based vapes will correlate with pulmonary injury and potentially disease, yet few larger-scale epidemiological studies have examined these effects in humans. Studies of airway inflammation typically use invasive techniques like blood draws, bronchial biopsies, or bronchoalveolar lavage. However, airway health can be assessed non-invasively from biospecimens like exhaled breath.

Breath analysis has gained acceptance mainly due to its potential ability to perform real-time assessment of volatile metabolites and ease of sample collection [15]. Breath is a complex chemical mixture from different sources, with its contents produced endogenously by biochemical processes or by microbiota, and from exogenous exposure. During respiration, chemicals are exchanged with the bloodstream at the alveolar interface, allowing hundreds of metabolites (peptides, lipids, nucleosides, etc.) to be expelled by exhalation [16]. Several studies have shown the ability of breath to allow disease detection [17], respiratory disease monitoring [18] or metabolomics and biomarker research (9), among others.

Specifically, analysis of exhaled breath condensate (EBC) has been validated for evaluating disease states, even in clinical settings [19, 20]. EBC is the collection of exhaled aerosol droplets that contain thousands of reported metabolites. Researchers have used EBC to assess inflammatory responses from airway diseases such as asthma [21], COVID-19 [22], or influenza infection [23]. Therefore, the analysis of exhaled breath condensate can be applied to assess impacts of vaping/smoking on airway health.

In this paper, we report an extensive chemical characterization of EBC from 254 individuals who smoke tobacco, use e-cigarettes, smoke marijuana, vape THC/CBD (cannabidiol), or use a combination of these habits, to those who abstain. We aim to discern and describe the unique toxic and inflammatory biomarker profiles of each group to provide valuable information related to the safety and implications related to the use of new generation smoking products. While the approach allows for the identification of key inflammatory biomarkers (oxylipins), it does not seek to establish direct links to clinical outcomes, such as lung disease or respiratory dysfunction.

## Methods

A brief description of the materials and methods used for this research are as written in this section. For a full description of materials and methods, reference Supplementary File 1.

### Study population and classification

This research followed clinical practices and protocols approved by the University of California, Davis Institutional Review Board (Protocol #1671798). Participants in this study were recruited primarily in university facilities, which may have influenced the demographic characteristics of the sample. A total of 254 participants were recruited and all participants were required to fill out a survey which inquired about usage and demographic information. Although this approach provides a broad picture of use, introduces potential biases, such as social desirability or inaccuracy in reporting.

Using the survey with the self-reported data, the groups were made as follow those who reported use of at least one tobacco or cannabis product within the past 30 days were considered active users, otherwise they were considered non-users for this study. Of the 254 participants, 132 reported to be users and 122 reported to be non-users. Subjects identified as users were classified into eight groups.

- **Group 1: Tobacco smokers** (those who reported smoking cigarettes, cigars, etc. in the past 30 days). *N* = 43
- **Group 2: Nicotine vapers/e-cigarettes users** (those who reported vaping nicotine via e-cigarettes, vapes, etc.). *N* = 46
- **Group 3: Users of nicotine in any form** (combustion and/or e-cigarettes, vapes). *N* = 70
- **Group 4: Marijuana smokers** (those who reported smoking cannabis flower via joints, bongs, etc.). *N* = 75
- **Group 5: THC/CBD vapers** (those who reported vaping THC and/or CBD). *N* = 63
- **Group 6: Users of cannabis, THC, or CBD in any form** (combustion and/or vapes). *N* = 108
- **Group 7: Users of any type of combustion-based tobacco and/or cannabis product** (cigarettes, joints, bongs, cigars, water pipes, etc.). *N* = 95
- **Group 8: Users of any type of electronic delivery device for nicotine and/or THC/CBD** (vapes, e-cigarettes, etc.). *N* = 82

### Exhaled breath condensate sampling and preparation

Exhaled breath condensate sampling uses a collection tool and methodology previously reported [24, 25]. In summary, subjects breathed tidally for 20 min through a disposable valved mouthpiece (no nose clip) connected to a trap that separates saliva and larger contaminants. The trap is attached to a glass tube surrounded by dry ice at − 80 °C to condensate exhaled aerosol droplets onto the interior surface of the tube. Subjects typically generate 1–2 mL of condensate, which is then retrieved from the tube and stored in at − 80 °C until chemical analysis.

The sample preparation applied was the same described in previous work in our group [22, 23, 26]. Briefly, samples were thawed and 1 mL of EBC (or the maximum amount available, if less than 1 ml) was aliquoted in 20 mL glass amber vial. An antioxidant solution and a mixture of internal standards were added to all samples. The antioxidant solution consisted of butylated hydroxytoluene (BHT) and EDTA at 0.2 mg mL^−1^ each in a solution of methanol:water (1:1). Internal standards consisted of a mixture of isotopically labeled (deuterated) oxylipins.

### Liquid chromatography–mass spectrometry (LC–MS) analysis of exhaled breath condensate

The metabolomic content of EBC was analyzed by liquid chromatography-mass spectrometry (LC–MS) using a simultaneous targeted and untargeted strategy, as it was described in previous work [22]. We targeted 55 oxylipins of inflammation and oxidative stress formed by lipid oxidation. Meanwhile, the untargeted approach used the entire range of detectable metabolites.

### Chemometric analysis

The data obtained from the untargeted approach was processed using the approach described in previously mentioned work [22]. Briefly, the chromatogram peaks were pre-process by an alignment and deconvolution process before removing the non-informative features coming from blank samples. Finally, potential breath markers were analyzed by PLS-DA and ranked by variable importance in projection (VIP) values, which summarizes the discrimination contribution of each feature makes to the model. Features with VIP scores higher than 1 are considered relevant markers for the corresponding consumer group.

## Results

### Study population

Table 1 details the number of participants for each studied group, highlighting the distribution according to our 8 main groups, defined in the “Materials and methods” section. Additional demographic information is found in Supplementary Table 1.

**Table 1:** Number of participants from the main consumer groups studied, including nonusers of any cannabis or tobacco product

User groups		Tobacco / Nicotine		Marijuana / THC		Total Users	Total Non-users
		Combustion	E-device	Combustion	E-device		
1	Tobacco smokers	43				43	122
2	E-cigarette vapers		46			46	122
3	Nicotine product users	70				70	122
4	Marijuana smokers			75		75	122
5	THC/CBD vapers				63	63	122
6	Cannabis product users			108		108	122
7	Combustion users	19 (+ 22)		54 (+ 22)		95	122
8	E-devices users		19 (+ 27)		36 (+ 27)	82	122

Participants were classified according to self-reported use. Values in parentheses indicate the number of participants who reported dual use

The cohort includes a balanced distribution between non-users, users of tobacco and cannabis products, and users of electronic devices. Participants from different self-reported race/culture/ethnicity were enrolled, for instance, Asian (30.7%), Latino/Hispanic (20.5%), African American (2.0%), White (24.8%), Multiracial (18.1%), Middle eastern (2.8%). Given that sampling carried out in university facilities participants were young adults ranging between 18 to 87 years of age, with an overall mean age of 28.6 ± 12.3 years, although this varied between groups (Supplementary Table 1).

Among the 254 participants in this study, 122 did not report use of any tobacco or marijuana related product in the last 30 days prior to the collection (described as non-users). Within this group, 98 declared no secondhand exposure to cannabis or nicotine products. However, 9 non-users reported regular exposure to both products at home or another setting, 10 indicated frequent exposure to cannabis related products at home, and the remaining 5 indicated frequent nicotine related exposure.

Cigarette users reported smoking on average 7.8 cigarettes in the past 30 days, varying between 1 and 50 cigarettes a day. Nicotine vapers or e-cigarettes users (Group 3) reported vaping an average of 11.6 of the 30 days prior to breath collection. Marijuana smokers (Group 2) reported the highest frequency of use, averaging 14 days of consumption within the previous 30 day period. Lastly, 63 participants (24.8% of the total) reported having vaped THC or CBD in the past month (Group 4).

Among the entire study population, 41 participants reported only one habit (10 only smoked cigarettes, 7 only used e-cigarettes, and 24 only marijuana). Reported co-use was more common: 42 individuals reported two habits simultaneously, 21 individuals used three habits, and 8 individuals (3.35% of the total) used four of the habits.

### Targeted analysis of inflammatory markers

We took a dual targeted and untargeted data approach to discover breath metabolites that correlate with tobacco and cannabis use habits. For the targeted analysis, we investigated 55 oxylipins of inflammation and oxidative stress formed by lipid oxidation. Mainly, we were interested in metabolites involved regulating inflammation from the cyclooxygenase and lipoxygenase pathway, as well as the cytochrome P450 pathway, critical in elimination of endogenous and exogenous compounds. Of our target molecules, 23 oxylipins were detected above the method limit of detection, and quantified.

To identify oxylipins that correspond to tobacco/cannabis consumption, eight PLS-DA models were built, comparing one user group against non-users. Table 2 summarizes the performance ability of PLS-DA models to classify users from non-users. Classification accuracies ranged from 0.64 ± 0.05 to 0.76 ± 0.05, suggesting a moderate ability for models to distinguish users based on oxylipins. Models from THC/CBD vapers (Group 4) and tobacco smokers (Group 1) resulted in the highest accuracies (both 0.76 ± 0.05). On the other hand, users of any type of cannabis product (Group 7) presented the lowest accuracy values, 0.64 ± 0.05.

**Table 2:** Performance of PLS-DA models to distinguish tobacco/cannabis users from non-users based on oxylipins and untargeted metabolites found in exhaled breath condensate

		Targeted oxylipin model performance						Untargeted metabolite model performance					
Groups		# Users	Non-users	Signif. Features	AUC	Sens	Spec	# Users	Non-users	Signif. Features	AUC	Sens	Spec
1	Tobacco smokers	38	120	13	0.76 ± 0.05	0.69 ± 0.04	0.71 ± 0.14	40	118	219	0.79 ± 0.06	0.72 ± 0.12	0.71 ± 0.06
2	E-cigarette vapers	52	120	15	0.68 ± 0.07	0.67 ± 0.08	0.54 ± 0.12	43	118	225	0.74 ± 0.07	0.59 ± 0.14	0.70 ± 0.10
3	Nicotine product users	60	120	15	0.72 ± 0.04	0.66 ± 0.06	0.69 ± 0.11	64	118	241	0.74 ± 0.05	0.66 ± 0.11	0.67 ± 0.07
4	Marijuana smokers	58	120	13	0.71 ± 0.07	0.66 ± 0.06	0.61 ± 0.16	61	118	268	0.77 ± 0.06	0.63 ± 0.13	0.73 ± 0.08
5	THC/CBD vapers	41	120	14	0.76 ± 0.05	0.67 ± 0.07	0.75 ± 0.16	54	118	264	0.75 ± 0.06	0.65 ± 0.13	0.68 ± 0.07
6	Cannabis product users	84	120	16	0.64 ± 0.05	0.68 ± 0.07	0.47 ± 0.09	90	118	268	0.74 ± 0.05	0.58 ± 0.09	0.75 ± 0.08
7	Combustion users	76	120	14	0.72 ± 0.04	0.67 ± 0.06	0.61 ± 0.11	81	118	284	0.79 ± 0.04	0.66 ± 0.10	0.75 ± 0.07
8	E-devices users	70	120	13	0.70 ± 0.06	0.69 ± 0.06	0.57 ± 0.10	73	118	269	0.73 ± 0.05	0.60 ± 0.08	0.70 ± 0.08

“Signif Features” show the number of compounds significantly impacted within a user group, relative to nonusers. AUC = Accuracy, Sens = Sensitivity, Spec = Specificity

Although the PLS-DA model obtained for Group 6, showed an AUC which is lower than the AUC obtained for Groups 4 and 5, this could reflect the slightly heterogeneity in Group 6. Because, while Group 6 includes individuals who exclusively smoke marijuana and exclusively use THC/CBD e-devices, along with those who combine these methods. While models for Groups 4 and 5 includes individuals who exclusively smoke marijuana or exclusively use THC/CBD vapes (respectively), along with those who combine these methods. Furthermore, differences in frequency, dose, and consumption methods may have also contributed to the limited discriminatory capacity of the Group 6 model. Future studies could refine group definitions or use more robust statistical approaches to improve discriminatory power.

It is important to note that even when the performance data such as AUC, sensitivity and specificity are relevant, the goal of using PLS-DA in this study was to identify the most discriminating compounds, defined as those with VIP values greater than 1.

**Fig. 1:**
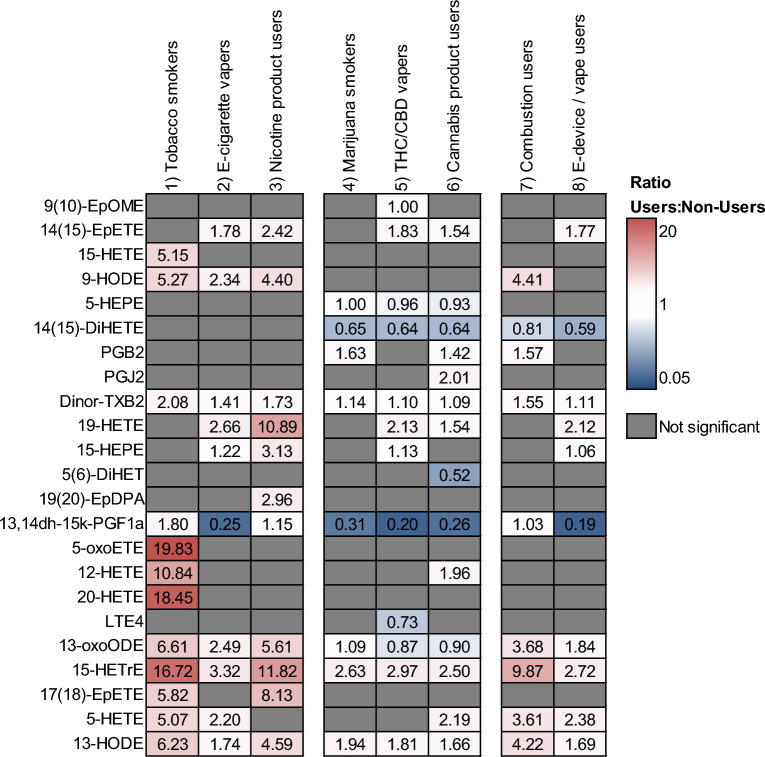
Of the targeted 55 oxylipins in exhaled breath condensate, these 23 were found to be significantly up- (red) or downregulated (blue) in users of tobacco and cannabis products, relative to non-users. Shown are the concentration ratios of users to non-users for significantly impacted compounds

Each model provided VIP scores for oxylipins, and those with a score > 1 were considered relevant. Compounds were additionally filtered through significance testing (p > 0.05) and with a fold change >|2|. Figure 1 shows the resulting oxylipins, presented as the ratio of the average concentration of users to nonusers. All 23 detected oxylipins were found to be significant for at least one user group.

### Oxylipins upregulated relative to nonusers

Overall, users of nicotine products showed more upregulated oxylipins than cannabis users (Fig. 1). Cigarette smokers exhibited the highest number of oxylipins, twelve, upregulated relative to nonusers, followed by nicotine product users (11 oxylipins) and e-cigarettes / vapes users (9 oxylipins). Marijuana smokers exhibited slightly fewer upregulated oxylipins: users of any cannabis product had 9 impacted compounds, whereas marijuana smokers and THC/CBD vapers both exhibited a total of 6 upregulated oxylipins.

Similarly, users of nicotine products showed a higher degree of upregulation relative to cannabis users (Fig. 1). Of the oxylipins found to be significantly higher, cigarette smokers had the highest average upregulation (average ratio to nonusers, “r”, r = 8.66) higher than nonusers, followed by users of any nicotine product (r = 5.17), then users of combustion products (r = 3.74), and e-cigarette / nicotine vapers (r = 2.13). Among cannabis users, THC/CBD vapers had a higher ratio of upregulated oxylipins, r = 1.83, followed by users of any cannabis product (r = 1.77) and marijuana smokers (r = 1.57). We observed tobacco smokers had the highest upregulation of any oxylipin: 5-oxoETE (r = 19.834), 20-HETE (r = 18.449), and 15-HETrE (r = 16.722). While 5-oxoETE and 20-HETE were only significant for tobacco smokers, 15-HETrE was significantly different for all user groups; however, for cannabis users, 15-HETrE was minimally upregulated relative to nonusers (r = 1.087), whereas 15-HETrE was downregulated in THC/CBD vapers (r = 0.871).

Users of combustion devices (tobacco and/or cannabis) showed a similar total number of upregulated oxylipins than users of e-devices, but combustion users showed higher upregulation. 9-HODE was higher (r = 4.41) in combustion users than nonusers but was not significantly different in users of e-devices. 14(15)-EpETE was higher in e-device users (r = 1.77), but not combustion users.

### Oxylipins downregulated relative to nonusers

Overall, there were fewer downregulated oxylipins among users, mostly occurring among users of cannabis. Tobacco smokers and nicotine product users did not have oxylipins downregulated relative to nonusers (Fig. 1), but e-cigarette / nicotine vapers had downregulation (r = 0.25) of 13,14dh-15k-PGF1a. E-cigarette users and users of any e-device (nicotine and THC/CBD) had on average the smallest ratios of significantly downregulated oxylipins, r = 0.25 and 0.39 respectively. This was followed by marijuana smokers, cannabis product users, and THC/CBD users (r = 0.48, 0.65, and 0.73).

13,14dh-15k-PGF1a was significantly downregulated in all user groups except those that included combustion of tobacco, in which it was significantly upregulated. 14(15)-DiHETE was downregulated in all cannabis users, but not significantly different among tobacco users. 9(10)-EpOME and LTE4 were downregulated only in THC/CBD vapers.

### Discussion of impacted oxylipins

Users of any type of tobacco product observed higher numbers of upregulated oxylipins and higher degrees of upregulation, with only 1 downregulated oxylipin unique to e-cigarette users. These findings are consistent with previous studies showing smoke tobacco exposition induces pronounced inflammatory signs in murine model, measure in bronchoalveolar lavage samples [27], it also affects the oxylipins profile, with high levels of proinflammatory oxylipins and other oxygenated lipids in plasma and bronchoalveolar lavage [28]. It is consistent with previously conducted studies that documented significant increases in proinflammatory oxylipins up to four-fold in plasma in smoking subjects with peripheral artery disease compare with non-smoking group, and a less inflammatory profile was described after the cessation [29].

On the other hand, cannabis users had fewer upregulated oxylipins and less upregulation overall, and had higher numbers of downregulated compounds, likely a consequence of the presence of cannabinoids that modulate the immune response despite the presence of reactive oxygen species [6]. Cannabinoids have demonstrated anti-inflammatory effects by suppressing proinflammatory cytokines in bronchoalveolar lavage and reducing oxidative stress in lung tissue, mechanisms mediated in part through the adenosine A2A receptor pathway in murine models [30]. Along with the cannabinoids compounds such as cannabis terpenoids have shown an important role in attenuating oxidative stress by inhibiting inflammatory pathways in murine lung epithelial cells [31, 32]. Furthermore, CBD reduces oxidative damage in murine pulmonary hypertension models in lung tissues, which could explain the oxylipin response observed in cannabis users [33].

Specifically, the up-regulation found by e-devices users was mainly caused by 14(15)-EpETE and 19-HETE, both compounds metabolized by the cytochrome P450 pathway, suggesting their activation by specific components of vaping liquids. Furthermore, the up-regulation of 9(10)-EpOME, in THC/CBD vapers, involves the same enzymatic activation (cytochrome P450).

This pattern contrasts with in tobacco smokers, where up-regulated 15-HETE, 5-oxoETE, and 20-HETE, are compounds more related to lipoxygenase activity, and would suggest a relationship with nicotine use by combustion.

Cannabis product users (including smokers and vapers) activated the lipoxygenase pathway, showed by 5-HEPE and 14(15)-DiHETE, and highlights the influence of cannabis on oxylipin profiles and differences with nicotine users [6].

Five oxylipins were significantly impacted among all user groups studied: Dinor-TXB2, 13,14dh-15k-PGF1a, 13-oxoODE, 15-HETrE, and 13-HODE. These results suggest an activation mainly of the cyclooxygenase and lipoxygenase pathway, regulating inflammatory processes and potentially influencing platelet reactivity (Dinor-TXB2), immune responses (13-oxoODE, 15-HETrE), as well as atherogenesis (13-HODE) [34]. Within a compound, the degree of up- or down regulation varied by user type, with higher ratios observed among tobacco users and lower ratios observed among cannabis users.

In contrast, the presence of 9(10)-EpOME and LTE4 in THC/CBD Vapers, would reflect an influence on the cytochrome P450 pathway and lipooxygenases, which are related in certain circumstances to respiratory pathologies and modulation of immune responses [35]. As for tobacco smokers, oxylipins such as 15-HETE, 5-oxoETE, 20-HETE, and 17(18)-EpETE highlight the involvement of arachidonic acid metabolism through the cytochrome P450 and 15-lipoxygenase pathways.

These findings suggest the need for public health campaigns considering the nuanced risk between different consumption methods, and marijuana combustion from tobacco combustion. However, given the differences in the profile of immune modulators (cannabinoids/terpenoids) present in the commercially available cannabis products [36] along with the wide range of potential benefits suggested in the literature [37], more studies should be performed to assess the airway inflammation impact between marijuana smokers and THC/CBD vapers. Furthermore, regulating and monitoring controls on cannabinoid concentration in vape liquids could help to assess their effects on airway health.

The findings of this study highlight the potential of exhaled breath condensate (EBC) analysis as a non-invasive tool for monitoring pulmonary health. Specifically, we observed a decrease in oxylipins associated with cannabis consumption and an increase in nicotine/tobacco use. These variations suggest that EBC profiling could serve as an exposure indicator of different substances as a proactive monitoring approach in the early status of chronic diseases. Policymakers could integrate EBC analysis into public health programs aiming at identification of at-risk populations and tailoring interventions to prevent or mitigate chronic respiratory diseases.

The bias toward a younger population reflects more prevalent consumption patterns in young adults, especially for cannabis and electronic devices, but limits the generalizability of the findings to older age groups. Furthermore, although our study population includes diversity in terms of gender, age, and ethnicity, some groups are underrepresented. These representation biases have different impacts on certain user subgroups. Furthermore, self-reported data can introduce recall or social desirability bias and even when our large cohort provides robust statistical power, these imbalance affects the applicability of the findings to the general population.

### Untargeted metabolomic analysis

In addition to targeting oxylipins, we employed an untargeted metabolomic approach to assess a wide range of potential compounds that provide extensive and exploratory view of possible toxicants profiles and metabolic differences among user groups. A total of 894 variables (features) were detected from exhaled breath condensate samples.

**Fig. 2:**
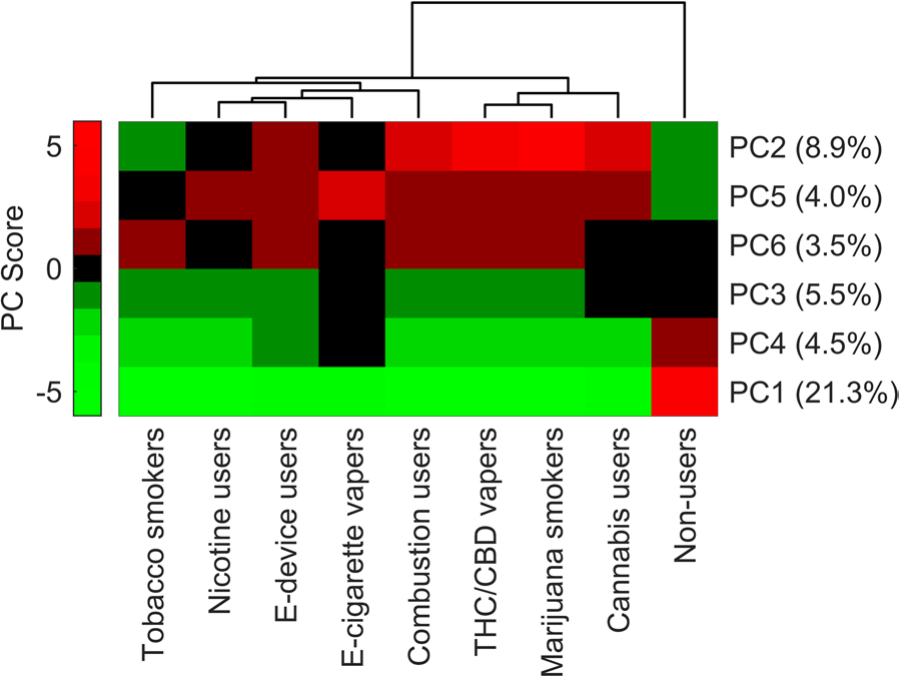
Hierarchical clustering analysis based on untargeted metabolite abundances compressed into six principal components (PC 1–6, shown with percentages of variance). The dendrogram shows non-users had a distinct breath metabolite profile than users, and that users of any type of cannabis product clustered separately from those using tobacco or nicotine. Our results suggest these habits have varying impacts on pulmonary metabolism

To reduce the number of variables, PCA was utilized with six principal components (PCs) selected. A hierarchical clustering analysis (HCA) was conducted on the six components, shown in Fig. 2. The dendrogram shows that non-users have a distinct metabolite profile from users of any type, likely driven by the differences in PCs 1, 2, 4, and 5, which sum to 38.7% of the total variance. Within the clusters of users, consumers of cannabis/THC had a distinct breath metabolite profile compared to those consuming tobacco.

PLS-DA models were built for each user group compared to non-users, with the results reported in Table 2. Overall, the untargeted results had slightly higher accuracies to discriminate users from non-users, relative to the targeted oxylipin analysis. Tobacco smokers and users of any combustion products showed the greatest discrimination with respect to non-users, indicated by the highest AUC values (0.79 ± 0.06 and 0.79 ± 0.04, respectively). Vapers (nicotine and/or THC) had the lowest accuracy, 0.73 ± 0.05.

Of all measured metabolites, 403 had a p-value < 0.05, fold change >|2|, and VIP > 1 relative to non-users. Of these, 275 metabolites were identified by comparison of the obtained experimental mass spectra to metabolomic libraries. Putative names, chemical class information, chemical formulae, and statistical values are listed in Supplemental Table 2.

**Fig. 3:**
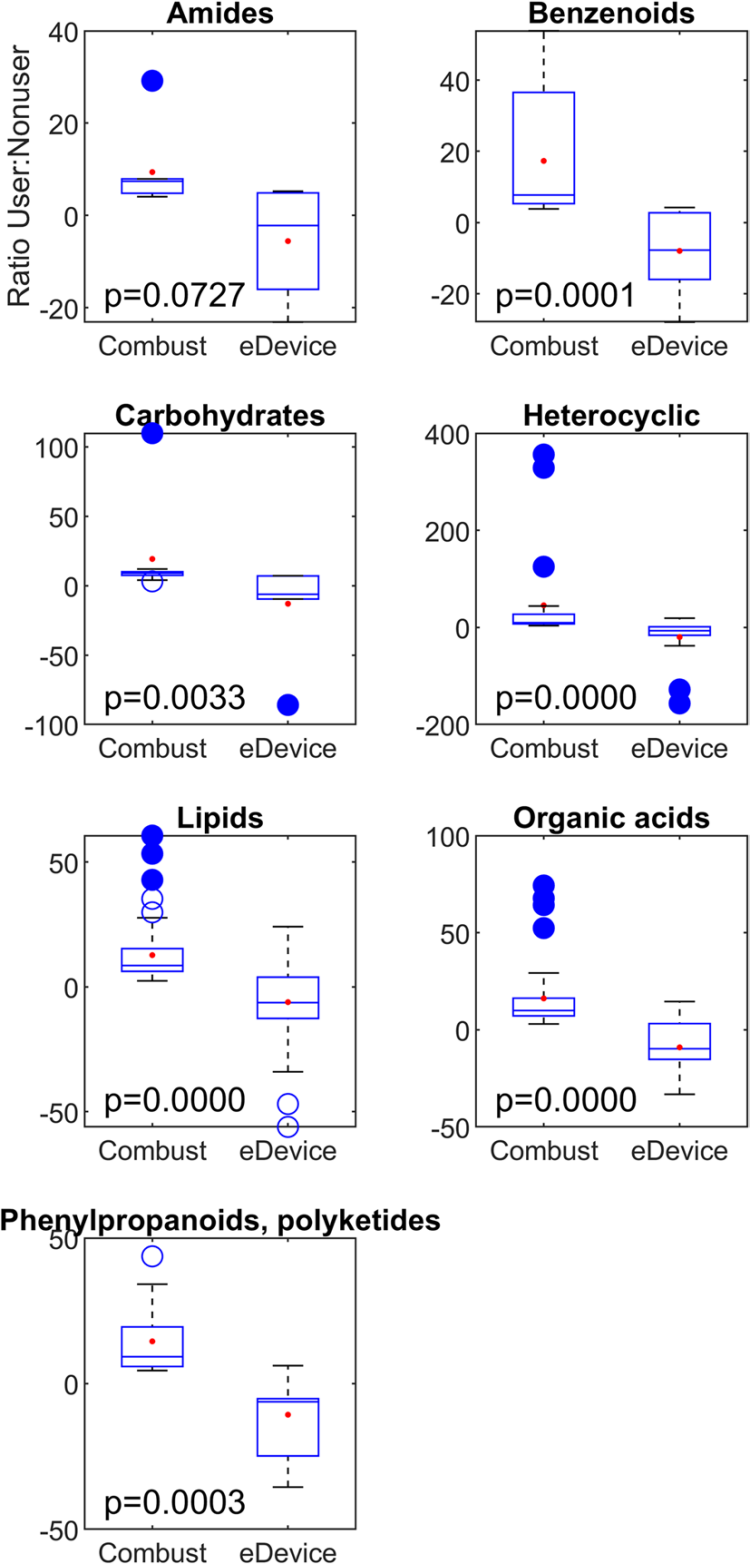
Boxplots comparing ratios of metabolites between combustion product users to nonusers (tobacco or cannabis), and e-devices users to nonusers (THC or nicotine), with corresponding p-values from Wilcoxon ranksum tests. Overall, metabolites were typically found in higher abundances among those who smoked products, relative to nonusers, and were found in lower abundances among e-device users. See also Supplemental Table 2

There were 8 major chemical superclasses described in all the breath samples. Interestingly, 7 of these superclasses had significance differences of the fold change ratios of users of any combustion product (tobacco, cannabis) and users of e-device products (vapes, e-cigarettes), both relative to non-users (Fig. 3). Metabolites were found in higher abundances in breath of people using combustion products, relative to nonusers, and found in lower abundances in people using e-delivery systems.

## Conclusions

Analysis of exhaled breath condensate was used to compare human metabolomic information of persons using tobacco and cannabis related products. Targeted measurements of oxylipin inflammatory markers found significant up-regulation among those using tobacco products relative to nonusers. Cannabis users exhibited oxylipin levels closer to and often downregulated compared to nonusers. However, direct links to clinical outcomes such as lung disease or respiratory dysfunction were not established, limiting conclusions about the clinical impact of these biomarkers.

Untargeted screening of breath metabolites found that users of cigarettes, nicotine vapes, and any tobacco product had similar metabolite profiles, whereas cannabis smokers, vapers, and product users had a profile that was more similar to nonusers.

However, the demographic characteristics of the sample, influenced by recruitment in university settings and the reliance on self-reported data to measure substance use introduce additional uncertainty, limiting the generalizability of the results to the general population.

## Supplementary Information

Additional file 1.

Additional file 2.

Additional file 3.

## Data Availability

Data is provided within the manuscript or supplementary information files.
