# Supplementary material for: Impacts of vaping and marijuana use on airway health as determined by exhaled breath condensate (EBC)
Source: Respir Res. 2025 Feb 21;26:63. doi: 10.1186/s12931-025-03147-3 (PMC11846476; doi:10.1186/s12931-025-03147-3)
Supplement: Supplementary file 1 — Additional file 1. [file 12931_2025_3147_MOESM1_ESM.docx]

|  | **Non-users** | **Users** | **Tobacco smokers** | **E-cigarette vapers** | **Nicotine product users** | **Marijuana smokers** | **THC/CBD vapers** | **Cannabis product users** | **Combustion users** | **E-device users** |
| --- | --- | --- | --- | --- | --- | --- | --- | --- | --- | --- |
| **N** | 122 | 132 | 43 | 46 | 70 | 75 | 63 | 108 | 95 | 82 |
| **Age (Avg ± SD)** | 32.9 ± 17.7 | 28.2 ± 12.1 | 34.7 ± 13.6 | 26 ± 8.9 | 30.2 ± 12.4 | 26.2 ± 7.8 | 25.1 ± 6.3 | 26.8 ± 10.5 | 28.8 ± 11.2 | 25.7 ± 7.8 |
| **Female** | 64 | 66 | 19 | 21 | 32 | 38 | 31 | 53 | 46 | 44 |
| **Male** | 58 | 66 | 24 | 25 | 38 | 37 | 32 | 55 | 49 | 38 |
| **Race / Ethnicity:** | | | | | | | | | | |
| **Asian** | 42 | 36 | 11 | 15 | 20 | 18 | 19 | 30 | 24 | 24 |
| **Latino / Hispanic** | 31 | 21 | 4 | 6 | 7 | 16 | 11 | 20 | 16 | 14 |
| **White** | 29 | 34 | 17 | 11 | 23 | 17 | 12 | 22 | 26 | 18 |
| **African American / Black** | 2 | 3 | 1 | 2 | 2 | 1 | 3 | 3 | 1 | 3 |
| **Middle Eastern** | 3 | 4 | 3 | 2 | 3 | 0 | 0 | 1 | 3 | 2 |
| **Multi Racial** | 13 | 33 | 7 | 9 | 14 | 22 | 17 | 31 | 24 | 20 |
| **Other** | 1 | 1 | 0 | 1 | 1 | 1 | 1 | 1 | 1 | 1 |
| **N/A** | 1 | 0 | 0 | 0 | 0 | 0 | 0 | 0 | 0 | 0 |

**Supplemental Table 1.** Demographic description of participants.
