# Supplementary material for: Impacts of vaping and marijuana use on airway health as determined by exhaled breath condensate (EBC)
Source: Respir Res. 2025 Feb 21;26:63. doi: 10.1186/s12931-025-03147-3 (PMC11846476; doi:10.1186/s12931-025-03147-3)
Supplement: Supplementary file 3 — Additional file 3. [file 12931_2025_3147_MOESM3_ESM.docx]

**Materials and Methods Supplementary File 1**

## **Study population and specimen collection**

This research followed clinical practices and protocols approved by the University of California, Davis Institutional Review Board (Protocol #1671798). Informed consent was provided by each volunteer prior to participating in the study. We recruited 254 participants aged 18+ from the UC Davis campus and surrounding Sacramento area (California, US).

Participants completed a nine-page survey to collect: demographic information (reported in **Supplementary Table 1**); their self-reported use and frequency of tobacco and cannabis consumption, including combustion and electronic delivery systems; and a brief medical history and recent medication use. Subjects then provided a sample of their exhaled breath condensate, described below.

## **Subject classification into user groups**

Subjects were classified as users if they self-reported nicotine or cannabis use in the 30 days prior to sample collection; otherwise, they were considered non-users, regardless of their history of use. There was a total of 122 non-users in the study cohort.

It is important to consider that very few of the remaining 132 participants reported using only one type of combustion or vaping product; by far, most users reported a mixed use of products. This complexity led to an adaptive classification strategy, where individuals with varied consumption habits were assigned to multiple groups according to their specific practices. However, the analysis methodology was designed to compare groups of users against the group of non-users. This approach sought to highlight the distinct impact of each consumption modality, while recognizing the potential overlap of effects among users.

The 132 “user” participants were separated into the following eight groups. Some users fell into multiple categories; see also **Table 1** for clarification.

- **Group 1: Tobacco smokers** (those who reported smoking cigarettes, cigars, etc. in the past 30 days). *N*=43
- **Group 2: Nicotine vapers/e-cigarettes users** (those who reported vaping nicotine via e-cigarettes, vapes, etc.). *N*=46
- **Group 3: Users of nicotine in any form** (combustion and/or e-cigarettes, vapes). *N*=70
- **Group 4: Marijuana smokers** (those who reported smoking cannabis flower via joints, bongs, etc.). *N*=75
- **Group 5: THC/CBD vapers** (those who reported vaping THC and/or CBD). *N*=63
- **Group 6: Users of cannabis, THC, or CBD in any form** (combustion and/or vapes). *N*=108
- **Group 7: Users of any type of combustion-based tobacco and/or cannabis product** (cigarettes, joints, bongs, cigars, water pipes, etc.). *N*=95
- **Group 8: Users of any type of electronic delivery device for nicotine and/or THC/CBD** (vapes, e-cigarettes, etc.). *N*=82

## **Exhaled breath condensate sampling and preparation**

Exhaled breath condensate sampling used a collection tool and methodology previously reported [24, 25]. In summary, subjects breathed tidally for 20 min through a disposable valved mouthpiece (no nose clip) connected to a trap that separates saliva and larger contaminants. The trap is attached to a glass tube surrounded by dry ice at −80 °C to condensate exhaled aerosol droplets onto the interior surface of the tube. Subjects typically generate 1-2 mL of condensate, which is then retrieved from the tube and stored in at −80 °C until chemical analysis.

The sample preparation applied was the same described in previous work in our group [22, 23, 26]. Briefly, samples were thawed and 1 mL of EBC (or the maximum amount available, if less than 1 ml) was aliquoted in 20 mL glass amber vial. An antioxidant solution and a mixture of internal standards were added to all samples. The antioxidant solution consisted of butylated hydroxytoluene (BHT) and EDTA at 0.2 mg mL^−1^ each in a solution of methanol:water (1:1). Internal standards consisted of a mixture of isotopically labeled (deuterated) oxylipins.

Deriving from exhaled aerosols, EBC may contain potentially infectious agents like SARS-CoV-2, and for this reason an inactivation step was done by adding acetonitrile and water (9 mL and 10 mL, respectively), followed by 15 min of incubation [27]. Acetonitrile was then evaporated under nitrogen stream at room temperature, and the remaining water was lyophilized until completely dried. Dried extracts were reconstituted with 60 µL of mobile phase (95% water in acetonitrile), vortexed, sonicated for 10 min at 4 °C and centrifuged at 13,000 rpm for 10 min at 4 °C. The supernatant was stored at −80 °C until mass spectrometry analysis, described below.

Pooled quality controls (QCs) were also prepared with each batch of samples using non-users subjects and spiking it with known concentrations of oxylipins.

## **Liquid chromatography – mass spectrometry (LC-MS) analysis of exhaled breath condensate**

The metabolomic content of EBC was analyzed by liquid chromatography-mass spectrometry (LC-MS) using a simultaneous targeted and untargeted strategy. We targeted 55 oxylipins of inflammation and oxidative stress formed by lipid oxidation. Mainly, we were interested in metabolites involved in cyclooxygenase (e.g. prostaglandins and thromboxanes), lipoxygenase (e.g. 5-, 12-, 15-HETE, leukotrienes, DiHETEs, HEPE, etc), and cytochrome P450 (e.g. HETEs, EETs, DiHOMEs, etc) pathways. We analyzed a balance of pro- and anti-inflammatory oxylipins previously reported in clinical investigations of asthma [21] using a tandem MS/MS instrumentation approach. Meanwhile, the untargeted approach used the entire range of detectable metabolites.

EBC samples were analyzed with an Agilent 1290 series HPLC system coupled with an Agilent 6530 quadrupole-time of flight (qTOF) mass spectrometer (Agilent Technologies). Twenty micro liters of sample were injected into the column using an autosampler at 5 °C. An InfinityLab Poroshell 120 EC-C_18_ column (3.0 mm × 50 mm, 2.7 µm, Agilent Technologies) held at 35 °C was used to separate compounds at a flow rate of 600 µL min^−1^. Compound separation was achieved using gradient of water (solvent A) and acetonitrile (solvent B), both with 0.1% formic acid during a total run time of 30 min.

An Agilent Jet Stream nebulizer was used as electrospray ionization (ESI) source. Samples were analyzed twice, one in positive and one in negative ESI mode, with mass ranges of 60–1000 and 100–970 m/z for positive and negative, respectively. It was operated at 250 °C with ionization set at 3000(+)/4000(−) V and fragmentor voltage at 130 V. Nebulizer gas pressure, temperature and drying gas flow rate were set at 45 psi, 400 °C and 10 L min^−1^. Mass measurements were recalibrated using Agilent reference masses m/z 121.0508 and 922.0098 in positive mode, and m/z 119.0363 and 966.0007 in negative mode. In both cases, all ions MS/MS acquisition was performed at collision energies of 0 and 15 V. Mass spectra were acquired at a scan rate of 2 spectra/s.

## **Chemometric analysis**

The data analysis process was divided into two strategies: targeted and untargeted approaches. Targeted analysis focused on markers of airway inflammation while the untargeted strategy focused on toxins and markers of oxidative stress. Targeted and untargeted information were acquired simultaneously in a single LC-MS run from the ESI negative mode injection of each breath sample; the corresponding ESI positive mode injection was treated for only untargeted purposes. Post hoc, the ESI positive and negative untargeted data were merged into one dataset. Further described in the supplementary

For targeted purposes, data was treated with Agilent’s Mass Hunter Quantitative (qTOF) Analysis B.07.00 software, and compounds were identified, confirmed, and integrated using accurate mass, retention time and MS/MS information. All detected compounds were quantified using standard calibration curves and correcting their responses by corresponding surrogates. QCs were quantified to determine the validity of the calibrations.

For untargeted analysis, data were deconvoluted, aligned and integrated using Agilent’s Mass Hunter Profinder B.08.00 software. Initial peaks were found using 30 ppm and 0.025 Da mass tolerance, and retention time window of 0.3 min. Generated data (.pfa) was imported to Agilent’s Mass Profiler Professional (MPP, V13.0) software for re-alignment and initial identification. MPP generates datasets used for statistical purposes, with samples in columns and variables (called features) in rows, like targeted data, but containing peak areas instead of concentrations. To obtain reliable data, samples were randomly injected on the LC-MS, and QCs and IS signals were checked for a correct data analysis.

Raw datasets were cleaned by removing features that either appear in blank samples with signals higher than 10 (breath:blank ratio), are not detected in more than 50% of samples, have low repeatability (RSD >25% in the QC pooled samples), or have near-constant values (RSD <5% in all samples). Data were normalized to remove systematic bias between sample measurements using different approaches: by specific factor, such as sample volume, IS area, or combination of volume/IS, and by sum or median values of all features for each sample. All missing values were also replaced by the LOD/5 or by minimum positive value divided by 5, in targeted and untargeted data, respectively. Final datasets were log transformed to correct data heteroscedasticity [28].

Targeted and untargeted datasets were analyzed using univariate and multivariate techniques using Excel, MATLAB R2024a and PLS Toolbox V8.6.2 software. First, descriptive univariate analyses were performed by comparative analyses of means to assess the significance of the changes using fold change (FC) and parametric/non-parametric tests like t-tests/Wilcoxon rank sum test, or ANOVA/Oneway Kruskal–Wallis test. These tests allow to determine features that explain the groups aimed to differentiate by using Volcano Plots, where features with p-values less than 0.5 (FDR adjusted values Bonferroni correction) and FC values higher than 2 show statistical difference. An initial variable selection was applied on untargeted datasets using univariate methods. Then, multivariate models were used for comparative analyses. Principal component analysis (PCA) is initially used to obtain an overview of large datasets, visualize similarities and differences between observations, detect potential outliers and gain information about the metabolite features responsible for the observed patterns. Partial least-squares discriminant analysis (PLS-DA) is a classification method, where PLS models the correlation between the dataset of features and a matrix of responses that contains sample information and classes/groups. [29, 30]. Sensitivity (probability of correctly detecting a condition), specificity (ability to reject a condition) and area under the curve (AUC) values of the receiver operating characteristic (ROC) curves were defined as parameters to describe model results. AUC measures the classification problems at different thresholds, telling how much a model is capable to distinguish classes or groups [31].

PLS-DA provides a list of potential markers related to condition and other relevant biological information, ranked by variable importance in projection (VIP) values, which summarize the discrimination contribution of each feature makes to the model. Features with VIP scores higher than 1 are considered relevant markers for the corresponding consumer group. Identification of relevant markers was performed searching through METLIN database and using accurate mass information to calculate a molecular formula and a compound name for each marker. Matching experimental and theoretical spectral pattern (scores) compounds were tentatively characterized when score values were higher than 70%. Identified compounds were classified according to their chemical structure.
